# Development of Pharmacy Practice in European Countries—The Polish Perspective

**DOI:** 10.3390/pharmacy5030043

**Published:** 2017-08-02

**Authors:** Damian Świeczkowski, Piotr Merks, Natalia Cwalina, Miłosz J. Jaguszewski

**Affiliations:** 1 First Department of Cardiology, Medical University in Gdansk, Dębinki 7, 80-952 Gdańsk, Poland; ncwalina@gumed.edu.pl (N.C.); mjaguszewski@gumed.edu.pl (M.J.J.); 2 Department of Pharmaceutical Technology, Faculty of Pharmacy Collegium Medicum in Bydgoszcz, Nicolaus Copernicus University in Torun, Dr. A. Jurasza 2, 85-089 Bydgoszcz, Poland; piotrmerks@gmail.com

**Keywords:** pharmaceutical care, pharmacist, pharmaceutical law, pharmacy practice, community pharmacy

## Abstract

Polish pharmacy practice and the distribution of medicinal products in some European countries are still going through a significant transformation. Changes postulated by the pharmacists should strengthen their role, and the place of community pharmacies in the health care system in the context of—among others—the introduction and popularization of pharmaceutical care. Subsequently, these efforts may essentially ensure the professional independence of the pharmacists. The introduction of pharmaceutical care to the pharmacy practice in all European countries will help to improve the quality of patient care and treatment outcomes, and will lead to a better allocation of available resources. Herewith, we present an important voice in the international debate, showing the current changes in the pharmacy practice in Poland, a European Union (EU)-member from 2004. Indeed, this paper presents the perspective of the country in which the position of pharmaceutical care is not well-established, and the role of the pharmacist is still limited to dispensing medicinal products, more than decade after it joined EU.

## 1. Introduction

The role of the pharmacist in some European Union (EU) members (e.g., Poland) is still under development, and remains limited to dispensing medicines despite the continuing efforts of pharmacists and academic bodies [1]. Thus, we still observe the differences in the role of pharmacists and the place of community pharmacies between particular European countries. However, the recent proposed and introduced changes in the Polish pharmaceutical law should be considered as the next step in a long path to promoting innovative pharmacy practice into routine settings in an area where the implementation of pharmaceutical care is still in the primary phase. In this context, the amendments currently being discussed in Poland might be supportive for countries where the pharmacy profession is still under development. Among others, the discussion involved the concept of “pharmacy for pharmacist”, as well as an extensive effort to introduce new services to community pharmacies, which are understood as the real beginning of pharmaceutical care in Poland. Finally, we will be touching on different aspects mainly associated with the drug policy, e.g., free drugs above 75 years old or the idea of over-the-counter (OTC) drugs available only in the community pharmacy which also have a vital impact on the pharmacy practice. The aim of this paper is to present the Polish attempt to introduce the new regulations designed to change the drug policy. In this sense, our brief review contributes to the global discussion about the future of the pharmacy practice in Europe.

## 2. Community Pharmacy in Poland—The Legal Framework

The number of community pharmacies per 1,000 citizens in Poland remains relatively high, so logistical obstacles do not limit the access to pharmaceutical services [2]. Moreover, we observe the constant consolidation of community pharmacies including more than 50 facilities [3]. On the other hand, the role of the pharmacist is tied only to dispensing medications and/or providing little advice about dosage or how to properly store the drugs, although the pharmaceutical studies are conducted within the university setting [4,5]. Apart from pharmacists in the community pharmacy, we can identify pharmaceutical technicians who fulfilled 2-year training as well as supportive staff, with respective responsibilities defined by the separated legal acts [6]. Interestingly, in the past, almost everyone could be the owner of a community pharmacy. Nowadays, Polish legislation provides that only pharmacists may own and operate a pharmacy. The pharmaceutical law prohibits only the situation where physicians which are actively involved in their professional duties are the owner of the community pharmacy. Moreover, no more than 1% of community pharmacies can belong to one legal entity. Currently, every community pharmacy must be led by a manager with pharmaceutical education and a minimum of five years of experience in the community pharmacy or three years and specialization obtained during postgraduate education. The Pharmaceutical National Chamber is a professional self-governing body dedicated to caring for the profession and representing the community of pharmacists during the external negotiations [7].

## 3. Pharmaceutical Care in Poland—Still Not Implemented

Pharmaceutical services lead to an improvement in treatment effect, as one can see in the context of taking care of patients diagnosed with cardiovascular diseases. Numerous studies have shown that pharmaceutical care leads a reduction in blood pressure, an improvement in lipid profile, and better glycemic control, as well as—in a broader perspective—increasing the degree of patients’ adherence to recommended treatment [8,9]. The definition of pharmaceutical care had already been introduced to the Polish legal system in 2008, and was described as a documented process in which the pharmacist should work with the patient, the doctor and, if necessary, with other medical professionals in the optimization of pharmacotherapy [10]. Moreover, there are still many legal and organizational problems which prevent the implementation of effective pharmaceutical care to the Polish pharmaceutical practice, including; (i) disrupted flow of medical information between the doctor and the pharmacist; (ii) lack of separate places in community pharmacies which enable free and private conversation with the patient; and finally (iii) the difficulties associated with the access to confidential information, which undoubtedly includes those concerning patients’ state of health [11,12,13]. In the official document provided by the Pharmaceutical Care Section of the Polish Pharmaceutical Society which includes the strategy for Polish pharmacy, it is stated that effective implementation of pharmaceutical services will optimize pharmacotherapy, achieve more satisfactory outcomes, and improve the health-related quality of patients’ lives. The key part of this implementation continues to be the effective harmonization of new services with a different part of the health care system, which should lead to the situation where the circulation between data obtained from pharmaceutical services is easily transferred from community pharmacies to the general practitioner (GP) and vice versa. According to this statement, groups regarded as beneficiaries of pharmaceutical care are geriatric and chronically ill patients who were affected by polypharmacotherapy [14]. In the Polish health care system, the efficacy of clinical pharmaceutical care has so far been investigated in the context of hypertension. Skowron et al. showed a reduction of blood pressure in the study group covered by the pharmacist intervention based on the knowledge of hypertension, as well as the detection of drug-related problems with recommendations [15]. The implementation and popularization of pharmaceutical care and pharmacists’ greater activity in the context of the health care system in Poland may contribute to the rationalization of available resources. For instance, the introduction of repeat dispensing as one of the new services to community pharmacies can limit the number of patient consultations aimed at receiving new prescriptions from GPs [16]. In this context, it is worth noting that the health care system in Poland faces some system problems such as long waiting lists to see a specialist or have surgery and a persistent shortage of doctors and nursing staff [17]. Following this, nurses and midwives have recently received permission to prescribe some medicines that previously only doctors were authorized to prescribe, while the pharmacists still do not have similar right [18].

## 4. Reimbursed Services—The Future of Pharmacy Practice in Poland

The works on the concept of pharmaceutical care reimbursed from public funds gained momentum in August 2015 when the government established the Pharmaceutical Care Team at the Ministry of Health. Its task is to develop regulatory frameworks and specific legal solutions aimed at the introduction of advanced pharmaceutical services to everyday pharmaceutical practice. So far, the published information indicates that the actions of the above-mentioned team focus on two types of pharmaceutical services: giving advice to patients on the correct use of inhalers and medication review services. These services—at least in their initial stage of introduction—focus on elderly patients with chronic diseases and those affected by polypharmacy. The right to provide reimbursable services will be given to all pharmacies, which have signed a contract with the National Health Fund—an institution supervising the process of reimbursing health benefits from public funds. According to one of the concepts, a patient will have the right to choose one particular community pharmacy and will confirm this decision with a written declaration [19]. According to the unanimous opinion of all the parties, pharmaceutical care service should be reimbursable from public funds, and the patient’s participation in the costs should be marginal. The party which is most involved in the changes leading to an increase of the pharmacist’s role and greater importance of the place of community pharmacies in the Polish health care system remains The National Pharmaceutical Chamber, the body legally representing the demands of pharmacists in the negotiations, for example, with the government. At the beginning of September 2016, the Pharmaceutical Care Team was closed by ministerial decision. To best of our knowledge, so far, no official statement including the results of the working of the above-mentioned group has been publicly announced [20].

## 5. New Deal in Community Pharmacies—“Pharmacy for the Pharmacist”

At the beginning of April 2017, the President of Poland signed a new act which officially introduced the concept of “pharmacy for the pharmacist” [21]. This new act also brought forth other immediate changes to the pharmacy practice. In detail, the postulated act clarified that only a pharmacist or a partnership consisting solely of fully-qualified pharmacists can manage and control up to a maximum of four community pharmacies. According to the Polish Pharmaceutical Chamber, this number should allow for a direct and proper supervision of the community pharmacy setting, and consequently, improve patient safety. The new regulations also provided geographic limits for any newly-created community pharmacies. A new pharmacy could be opened when the number of people calculated per single pharmacy equals or exceeds 3000 individuals in a particular district and the distance from the planned location of the new pharmacy to the nearest accessible community pharmacy is at least 500 m [22,23]. “Pharmacy for the pharmacist” means that the main owner of community pharmacy should be a person with higher education and who has a license to practice a profession of a pharmacist in the Republic of Poland. In the opinion of the Polish Pharmaceutical Chamber (Supreme Pharmaceutical Council), this concept will lead to greater control over community pharmacies and strengthen the professional independence of the pharmacists [24]. The proposed changes are criticized by some organizations, which indicate that the legislation presented above may be unconstitutional, against European Union law, and cause an increase in the price of medicinal products [25,26]. Apart from the demands to introduce pharmaceutical care, currently there is also discussion on the concept of a separate act—The Act on the Profession of Pharmacist—which shall be a summary and collection of so-far scattered regulations.

## 6. OTC Drugs—Only in Community Pharmacies

It is worth mentioning the demands of the restriction of the non-pharmacy distribution of drugs; at present, some of the medicinal products available OTC are also available in convenience stores or petrol stations and are dispensed by people without even basic pharmaceutical education [27]. These proposals are subject to intense criticism from organizations that bring together representatives of chain pharmacies. The proposed changes are contrary to the provisions of economic freedom, and may be in conflict with the Constitution of the Republic of Poland—a central piece of legislation in Poland with which other adjustments must be compliant [28].

However, it should be noted that in the EU we do not identify a unified drug policy in the context of the availability of OTC medicinal products in the non-pharmacy distribution, which is consistent with the freedom of lawmaking by the members of the European community. Consequently, we may find the countries where the OTC drugs might be placed only in community pharmacy settings, such as Germany or Austria. For instance, in Germany, only dietary supplements and some herbal or homeopathic products can be purchased outside pharmacies. We may meet the same regulations in Spain, France, or Greece. On the contrary, a different and more liberal approach can be found in Italy or Denmark. Nevertheless, the tendency in the EU is focused on reducing the non-pharmacy distribution of drugs, and most OTC medicinal products can be purchased mainly in community pharmacies [29,30].

## 7. Free Drugs for Patients above 75 Years Old

The government has also made an effort to alleviate the financial burden associated with the purchase of medicines by the elderly. Currently in Poland there is a complex system for the reimbursement of medicinal products from public funds, and the level of patient co-payments for drugs remains one of the highest in Europe. On 1 August 2016, a project was published containing the list of medicinal products which will be free for patients above 75 years of age. This document provides a list of 1129 medicinal products containing a total of 68 active substances related to the treatment of old-age-diseases, including cardiovascular diseases, neurological disorders (e.g., Alzheimer’s disease), or mood disorders (e.g., depression). It is estimated that these changes are expected to contribute up to ca. 82 million dollars savings for geriatric patients [31,32]. The primary objective of this project is to improve adherence. The patient’s high co-payment from their funds remains one of the causes of lower adherence [33]. However, it should be stressed that it does not solve the multifaceted problem of patient adherence. This is proved by the fact that the phenomenon of non-adherence also exists in countries such as the UK, where the majority of medicinal products are available free of charge for large groups of patients [34]. Therefore, the introduction of the list of free drugs for elderly patients should be considered a first step towards improving adherence and starting a debate on this issue.

## 8. Falsified Medicines Directive—New Responsibilities for Community Pharmacists

The implementation of EU regulations of the Falsified Medicines Directive (FMD) remains challenging and has been scarcely discussed so far [35]. Undoubtedly, the introduction of the FMD will lead to improvements in patient safety, but will also be associated with increasing staff demand as well as new responsibilities for community pharmacists [36]. Authentication will be the new and obligatory duty for pharmacists, which can be problematic in the Polish context due to the permanent lack of staff. Moreover, new software should be adequately introduced to avoid the possibility of errors in dispensing and to minimize disturbances in the workflow. It is worth mentioning that due to the legal proposals of FMD, the pharmacists will be able to detect falsified drugs easily and consequently remove them from the market [37]. In addition, the collection of safety alerts (e.g., adverse drug events) will also be more feasible [38].

All of these factors can lead to the conclusion that FMD will be beneficial in improving health care by the optimization of treatment. Indeed, the costs of its implementation should also be warranted and will not be limited to purchasing new software, but will be associated with the adjustment of community pharmacies to new requirements (e.g., logistics-related problems). The issue of the implementation of FMD in Polish pharmacies has already been succinctly discussed by other authors. Indeed, as a border country of the EU, Poland remains an important channel of illegal drug distribution and trade from “East to West”. Thus, the effectiveness of FMD implementation will be an integral part of the success of amendments proposed by the European Community, and in that sense, Polish pharmacists hold significant responsibilities for patient safety in the whole of Europe [35].

## 9. Other Challenges and a Glimpse of the Future

**Figure 1:**
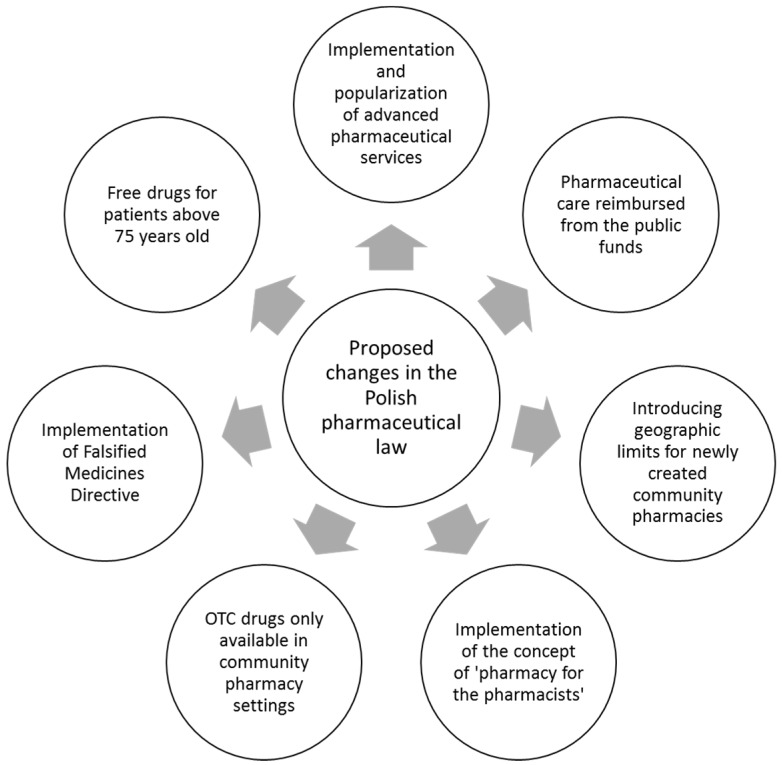
Proposed amendments in Polish pharmaceutical law—summary.

Obviously, our review does not cover all the topics which have recently become issues of discussion among Polish pharmacists. In addition, the introduction of the list of free medicines for elderly people also contributes to the return to the public debate about the problem of the lack of availability of certain medicinal products in community pharmacies (e.g., anticoagulants). This widely-discussed topic was one of the reasons for the introduction of regulations tightening the export of medicines outside the territory of Poland and even sanctioning this practice for some drugs—especially those which may not be accessible for Polish patients [39]. Moreover, another matter postulated by the pharmacists is to uphold the existing law which prohibits any form of advertising of pharmacies, including in the shape of loyalty cards entitling the patient to the promotional purchase of drugs [40]. The changes can also be seen in the undergraduate education of pharmacists; some faculties have introduced subjects more focused on clinical skills and aiming at deepening the pharmacists’ medical knowledge. Such classes often take place in university clinics, which allows students to have direct contact with the patient [41]. We summarized all amendments mentioned above in Table 1 and Figure 1. To sum up, the availability of community pharmacies—almost unlimited from the patient’s perspective—and the skills and knowledge of Polish pharmacists remain an untapped resource of the Polish health care system. Given the aging society and the observed worldwide trend of broadening the professional competence of pharmacists towards services optimizing pharmacotherapy and promoting pro-health education, the introduction of pharmaceutical care and legal changes in the distribution of the medicinal products in Poland should be considered highly necessary and useful. Without a doubt, Polish pharmacists are impatiently awaiting changes described in this comment and are ready for far-reaching changes in the pharmacy practice—Yes, we can!

## Figures and Tables

**Table 1:** Changes in the pharmacy practice in Poland—highlights.

Highlights
The introduction of pharmaceutical care to the Polish health care system will contribute to the consolidation of the pharmacist’s role and the place of pharmacies in Poland. Pharmaceutical care in Poland is to become a service reimbursable from public funds, particularly among patients with chronic diseases as well as the geriatric population. Implementation of the list of free-of-charge medicines for the elderly may lead to an improved adherence. However, this change should be the first step on the long path to solving the problem of non-adherence among Polish patients. The party most active in promoting the increasing role of the pharmacist in Poland remains The National Pharmaceutical Chamber. Some of the self-government’s demands are subject to intense criticism, especially in the context of the compatibility of the proposed amendments to the Constitution and the principle of economic freedom. An important challenge—to date discussed in the public debate to a small extent—is the implementation of provisions of the Falsified Medicines Directive (FMD) and the harmonization of national legislation with EU regulations into the Polish legal system.
